# Impact of tubewell access and tubewell depth on childhood diarrhea in Matlab, Bangladesh

**DOI:** 10.1186/1476-069X-10-109

**Published:** 2011-12-22

**Authors:** Jianyong Wu, Mohammad Yunus, Peter Kim Streatfield, Alexander van Geen, Veronica Escamilla, Yasuyuki Akita, Marc Serre, Michael Emch

**Affiliations:** 1 Department of Environmental Sciences and Engineering, Gillings School of Global Public Health, University of North Carolina at Chapel Hill, NC USA; 2 International Centre for Diarrhoeal Disease Research, Bangladesh; 3 Lamont-Doherty Earth Observatory of Columbia University, Palisades, NY USA; 4 Department of Geography, University of North Carolina at Chapel Hill, NC USA; 5 Carolina Population Center, University of North Carolina at Chapel Hill, NC USA

**Keywords:** Diarrheal disease, tubewell, groundwater, arsenic

## Abstract

**Background:**

During the past three decades in Bangladesh, millions of tubewells have been installed to reduce the prevalence of diarrheal disease. This study evaluates the impacts of tubewell access and tubewell depth on childhood diarrhea in rural Bangladesh.

**Methods:**

A total of 59,796 cases of diarrhea in children under 5 were recorded in 142 villages of Matlab, Bangladesh during monthly community health surveys between 2000 and 2006. The location and depth of 12,018 tubewells were surveyed in 2002-04 and integrated with diarrhea and other data in a geographic information system. A proxy for tubewell access was developed by calculating the local density of tubewells around households. Logistic regression models were built to examine the relationship between childhood diarrhea, tubewell density and tubewell depth. Wealth, adult female education, flood control, population density and the child's age were considered as potential confounders.

**Results:**

*Baris *(patrilineally-related clusters of households) with greater tubewell density were associated with significantly less diarrhea (OR (odds ratio) = 0.87, 95% confidence interval (CI): 0.85-0.89). Tubewell density had a greater influence on childhood diarrhea in areas that were not protected from flooding. *Baris *using intermediate depth tubewells (140-300 feet) were associated with more childhood diarrhea (OR = 1.24, 95% CI: 1.19-1.29) than those using shallow wells (10-140 feet). *Baris *using deep wells (300-990 feet) had less diarrheal disease than those using shallow wells, however, the difference was significant only when population density was low (< 1000 person/km^2^) or children were at the age of 13-24 months.

**Conclusions:**

Increased access to tubewells is associated with a lower risk of childhood diarrhea. Intermediate- depth wells are associated with more childhood diarrhea compared to shallower or deeper wells. These findings may have implications for on-going efforts to reduce exposure to elevated levels of arsenic contained in groundwater that is pumped in this study area primarily from shallow tubewells.

## Background

Diarrheal diseases are a major public health problem in the developing world. Approximately 1.5 million children die from diarrheal diseases each year globally, which makes it the second most common cause of mortality in children under five [1]. Diarrheal diseases can be attributed to contaminated drinking water, poor sanitation and hygiene, and more broadly to poverty [2,3]. In Bangladesh, diarrheal diseases are one of the leading causes of death in children under 5, accounting for 20% of all infant deaths [4]. In an effort to reduce diarrheal diseases, during the past 30 years Bangladesh has undertaken an almost universal shift from drinking surface water to drinking groundwater. The concentration of fecal indicator bacteria is typically orders of magnitude lower in groundwater compared to surface water in densely populated villages of Bangladesh [5]. Millions of tubewells have been installed and now provide drinking water for more than 95% of rural residents [6]. Diarrhea mortality has declined in the past four decades in rural Bangladesh, however, whether the decline in mortality can be attributed to tubewells is unclear [7-9], because other interventions have been carried out during the same time period [6].

During the 1990s, high levels of arsenic (As) were detected in shallow (typically less than 140 feet deep) aquifers in Bangladesh. According to a national survey conducted in the late 1990s [10], the As content of groundwater in one-third of the tubewells exceeded the Bangladesh permissible limit (50 μg/L) and did not meet the World Health Organization guideline of 10 μg/L in half of the tubewells. Exposure to this metalloid has a number of adverse health effects including cancers of the lung, liver, skin, and bladder as well as cardiovascular disease [11-13]. Before remediation efforts started to have a significant impact, between 35 and 77 million people were put at risk in Bangladesh due to the widespread contamination of groundwater with As [10]. The As crisis has forced policymakers to rethink the public health value of installing tubewells to avoid drinking microbially-contaminated surface water.

This study explores whether tubewell access reduces diarrheal disease risk and, if so, whether tubewells ≥ 300 ft deep are at least equally protective in terms of diarrheal diseases compared to shallow wells. This is an important issue because over 165,000 deep tubewells have been installed throughout Bangladesh to reduce exposure to As [14]. The relationship between diarrheal diseases in children under 5 and tubewell access and depth can be measured in this study because of the unique set of data that were collected over a 7 year period.

## Methods

### Study area

**Figure 1:**
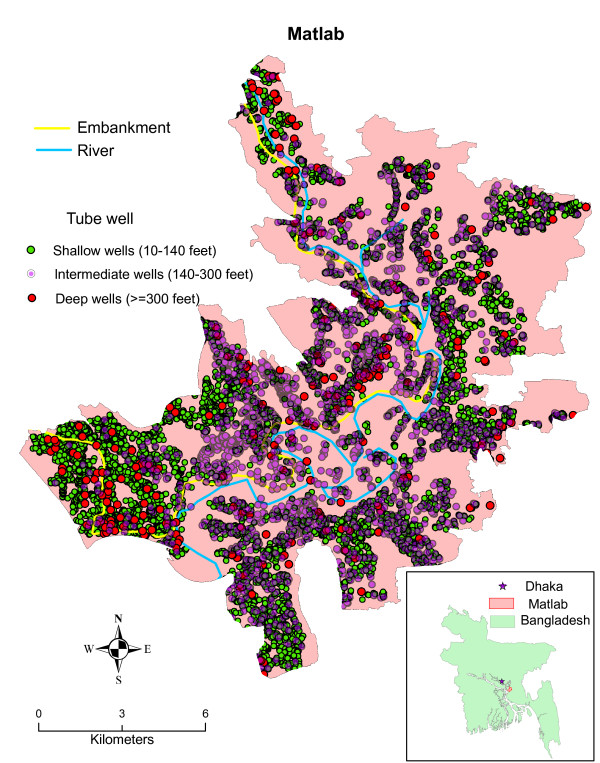
**The study area, Matlab, Bangladesh and the spatial distribution of tubewell depth**.

The study area is Matlab, Bangladesh, which is approximately 50 kilometers (km) southeast of Dhaka and is a field research site for the International Centre for Diarrhoeal Disease Research, Bangladesh (ICDDR, B). There are approximately 220,000 people living in the 142 villages of Matlab. Each village is composed of tens to hundreds of patrilineally-related clusters of households called *baris*. The area has an extensive Health and Demographic Surveillance System (HDSS) in which 120 community health research workers (CHRWs) visit each household every month to collect information on health and demographic events (Figure 1).

### Data collection

CHRWs collected diarrheal disease event data for under 5 year old children for the 10,945 *baris *in Matlab from 2000 to 2006. They asked parents if their children had diarrhea within the past 24 hour recall period. A diarrhea case was defined as at least three or more liquid stools in the past 24 hours. Cases were also categorized as either watery or bloody. Once a case was confirmed, the illness date, birthday, and gender of the child were recorded in the HDSS database. Since the annual number of cases is based on parental 24 hour recall on 12 visits per year, we use average daily cases (total cases in a year divided by 12) and average daily prevalence (average daily cases divided by the number of children) to estimate the magnitude of diarrhea in each *bari*.

**Figure 2:**
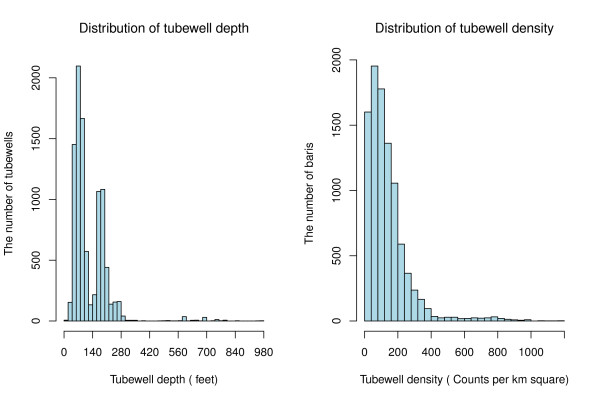
**The distribution of tubewell density and tubewell depth**.

A global positioning system (GPS) survey of all 12,018 tubewells in Matlab was conducted from February 2002 to August 2004. The depth of each tubewell was determined by asking the owners; they typically know how deep their tubewell is because the construction price is determined by the length of PVC pipe used for the installation. Tubewell depths range from 10 to 990 feet, with almost two-thirds of the wells between 10 and 140 feet deep, one third between 140 and 300 feet deep, and only 2.2% deeper than 300 feet (Figure 2).

A proxy for tubewell access was developed by calculating the local density of tubewells around *baris*. The number of tubewells within 100 meters of all *baris *was first calculated. A maximum radius of 100 meters was chosen because most *baris *had at least one tubewell within that distance. Three categories of roughly equal size were created based on the distribution of the tubewell density: (i) < 80 wells/km^2^, (ii) 80 to < 160/km^2^, and (iii) ≥ 160/km^2 ^(Figure 2). Each *bari *was given a density score ranging from 1 to 3 with higher scores representing a higher density of tubewells. Tubewells were also classified into three depth categories: (i) shallow wells (< 140 feet), (ii) intermediate-depth wells (140-300 feet), and (iii) deep wells (≥ 300 feet). The cutoff of 140 feet was chosen because there is a natural break at 140 feet in the depth distribution (Figure 2). The cutoff of 300 feet was chosen for deep wells because wells ≥ 300 feet deep require different and more expensive technology that is typically used only to install community wells paid for by the Government of Bangladesh or non-governmental organizations (NGOs) for arsenic mitigation.

Flood control, wealth, education, population density and the child's age were included in the analysis to determine if they alter the effect of access and depth on childhood diarrhea. A large flood control embankment was completed in 1990 which divides Matlab into a protected area with 4149 *baris *and unprotected area with 6796 *baris *[15,16]. Therefore, a flood control variable was created following a binomial distribution, namely, the flood control variable was coded as 1 if a *bari *was protected by the flood control embankment, and 0 otherwise. Previous studies have shown that flood control influences diarrheal disease incidence [15,17]. A categorical wealth variable was developed using principal components analysis [18] with 31% of the variance that is captured by the first principal component, creating a single household-level measure from multiple census variables [19]. The wealth measure reflects a composite of several variables of ownership of household assets (bed, bicycle, blanket, lamp, watch) and one ordinal variable of household wall material. Household-level wealth scores were then collapsed by *bari*, and the mean score represents the *bari*-level wealth. All *bari*-level wealth scores were sorted from lowest to highest and divided into five categories, with a large value reflecting a wealthier status. The adult female (age ≥ 15 years) educational status was measured in a census of Matlab in 2005. The educational status at *bari*-level was calculated by averaging the years of education of adult females in a *bari*. Then the years of education in *baris *was classified into 5 categories: (i) less than 1 year, (ii) 1 year to less than 3 years, (iii) 3 year to less than 5 years, (iv) 5 years to less than 7 years, and (v) 7 years and above. Population density was calculated using total population within 100 meters of *baris *divided by the area. It was then reclassified into three groups: 1 (0 < population density < 1000 person/km^2^), 2 (1000 ≤ population density < 3000 person/km^2^) and 3 (population density ≥ 3000 person/km^2^). The size of each group is comparable. The ages of children were calculated based on children' dates of birth. A *bari*-level child's age variable was created by selecting *baris *if all children in a *bari *are in same age, then the child's age was classified into five groups: 0-12 months, 13-24 months, 25-36 months, 37-48 months and 49-60 months.

A *bari*-level geographic information system (GIS) of the study area was created to link health and population data to particular *bari *locations including diarrheal disease events, population distributions, and the wealth scores and maternal education of households based on their geographic coordinates and identification numbers.

### Statistical analysis

*Bari*-level logistic regression models were built to examine the association between childhood diarrhea and tubewell access and depth. A binary dependent variable based on the average daily diarrhea prevalence was created. First, the average daily prevalence was created for the entire study area using the average daily cases divided by the total number of children under 5. The average daily prevalence of each *bari *(the average daily cases of a *bari *divided by the number of children in that *bari*) was then compared with the average daily prevalence of the entire area. If the prevalence of a *bari *was larger than the average it was assigned a value of 1 and if it was smaller it was assigned a value of 0.

Univariate logistic regression models were conducted first, followed by multivariate logistic regression models. In the univariate logistic regression models, either tubewell access or tubewell depth was used as the independent variable. The models for both independent variables also considered flood control, wealth, education, population density and the child's age as control variables. For multivariate logistic regression models, three control variables, flood control, education and population density were also put into the models as independent variables. We separated the wealth index from the education variable in the models because it is correlated with the education index (Spearman correlation coefficient r = 0.51, p < 0.0001, n = 49,475). When examining the relationship between tubewell depth and childhood diarrhea, *baris *drinking from shallow wells (< 140 feet) were the referent group which was compared to *baris *drinking from intermediate-depth wells (140-300 feet) and deep wells (≥ 300 feet), respectively. The association between childhood diarrhea and tubewell access and depth was indicated by an odds ratio (OR), which is the ratio of the odds of a one unit increment of the independent variables. Tubewell access was divided into 3 categories: low access = 1, medium access = 2 and high access = 3; tubewell depth also had 3 categories but they were modeled as dummy variables, namely, when shallow wells and deep wells were compared, shallow wells were given a value of 0 and deep wells a value of 1; when shallow wells and intermediate-depth wells were compared, shallow wells were given a value of 0 and intermediate-depth wells a value of 1. The 95% confidence intervals (CI) of the ORs are also reported. The logistic regression models were built using SAS 9.2 (SAS Inc., Cary, NC).

## Results

### Childhood diarrhea

**Figure 3:**
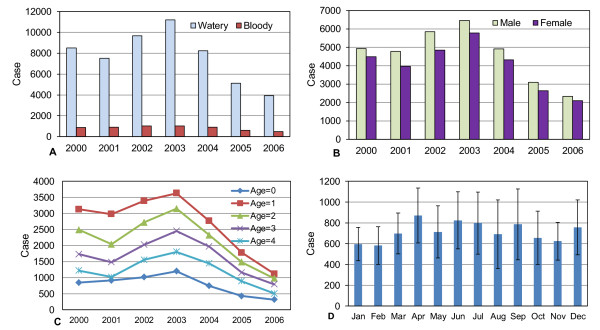
**Childhood diarrhea by type, gender, age, and season in Matlab, 2000-2006**. A: Watery and bloody diarrhea cases. B. Diarrhea cases by gender. C. Diarrhea cases by age. D. Diarrhea cases by month.

**Figure 4:**
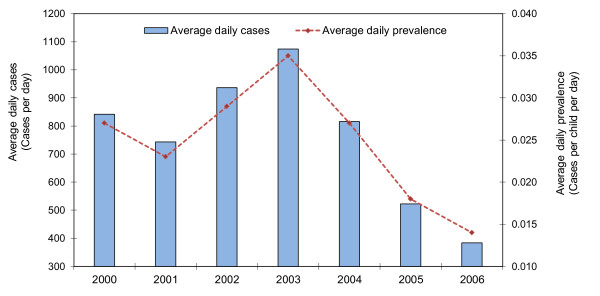
**The average daily cases and prevalence of childhood diarrhea in Matlab, 2000-2006**.

**Figure 5:**
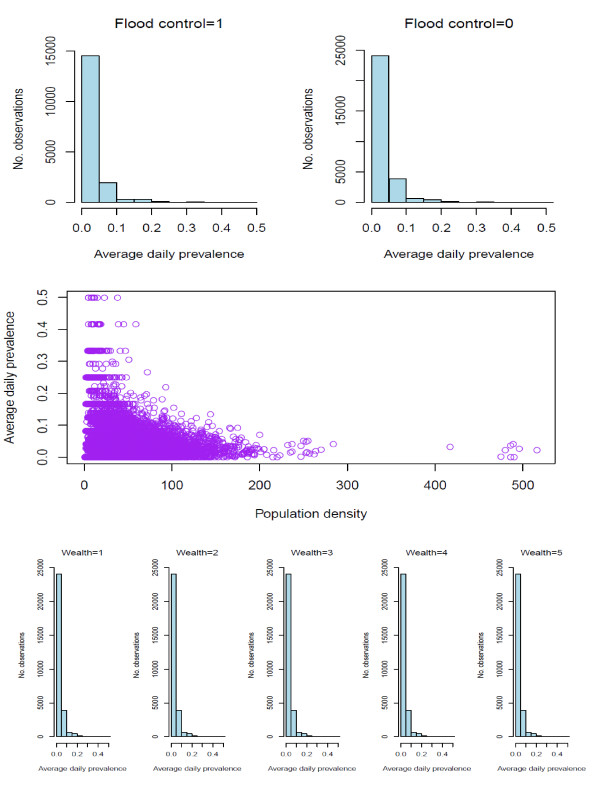
**The relationship between childhood diarrhea and flood control, population density and wealth**.

**Figure 6:**
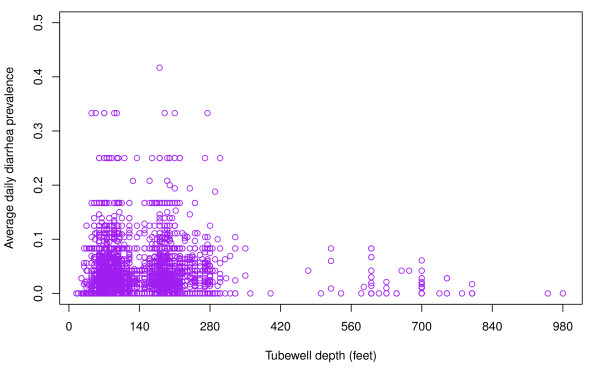
**Scatterplot of the relationship between average daily childhood diarrhea prevalence and tubewell depth**.

The distribution of childhood diarrhea cases by type shows that approximately 90% of cases are watery and 10% bloody diarrhea in each year (Figure 3A). Fifty-three percent of cases were in boys and 47% in girls (Figure 3B). In each year, infants less than 1 year had the lowest prevalence. The number of cases is highest among 1 year olds and then gradually decreases through age 4 (Figure 3C). There is no clear seasonal trend to childhood diarrhea during the study period (Figure 3D). The number of diarrhea cases during the 12 recall days each year was highest in 2003 (approximately 12,000 cases) and then gradually decreased through 2006 (approximately 4500 cases). The average daily prevalence was the highest in 2003 with 35 cases per 1000 children per day and the lowest in 2006 with only 14 cases per 1000 children per day (Figure 4). The distribution of cases of childhood diarrhea in relation to flood control, population density, and wealth variables is illustrated in Figure 5. The distribution of diarrhea was similar in flood controlled and unprotected areas, with ratios of diarrhea cases to population inside and outside of the embankment of 0.041 and 0.045, respectively. The distribution of diarrhea cases was also similar for groups with a different wealth status. The number of diarrhea cases increased with population density, however. The difference in diarrhea prevalence between shallow and intermediate-depth wells is difficult to discern from a scatter plot because of the large number of data points (Figure 6).

### Associations between childhood diarrhea and tubewell access

The univariate logistic regression model shows that greater tubewell access was associated with less childhood diarrhea (OR = 0.87, 95% CI: 0.85-0.89) (Table 1). The inverse relationship between childhood diarrhea and tubewell access was consistent in each year from 2000 to 2006, as all ORs and their 95% confidence intervals were significantly lower than 1 in each of the 7 years. The inverse relationship still holds when the models are controlled separately by wealth or population density. In each group of wealth status or population density, children in *baris *with higher tubewell access had a lower likelihood of diarrhea. With the exception of the group for which adult females received 7 or more years of education on average, tubewell access remained related to a lower likelihood of diarrhea. For *baris *with children less than 2 years old, the association between childhood diarrhea and tubewell access was not significant. The relationship between tubewell density and diarrhea was affected by the flood control variable. Outside the flood control area, the association was still significant (OR = 0.84, 95% CI: 0.82-0.87). However, there was no association between tubewell density and diarrhea prevalence within the flood control area (OR = 1.02, 95% CI: 0.98-1.06).

**Table 1:** Univariate analysis of the associations between childhood diarrhea and tubewell access in Matlab, 2000-2006.

Independent variable	Control variables	N	OR	95%CI		p
Density-based tubewell access	Unstratified	51406	0.87	0.85	0.89	< 0.001
	
	Flood control					
	
	Yes	17926	1.02	0.98	1.06	0.321
	
	No	33480	0.84	0.82	0.87	< 0.001
	
	Population density					
	
	Low	15048	0.87	0.83	0.91	< 0.001
	
	Medium	16919	0.85	0.82	0.89	< 0.001
	
	High	19439	0.81	0.78	0.84	< 0.001
	
	Wealth					
	
	Low	2797	0.94	0.85	1.04	0.242
	
	Low medium	13357	0.84	0.81	0.88	< 0.001
	
	Medium	21943	0.91	0.88	0.94	< 0.001
	
	High medium	10536	0.87	0.83	0.92	< 0.001
	
	High	2039	0.89	0.79	1.00	0.055
	
	Education					
	
	< 1 year	3862	0.90	0.82	0.98	0.018
	
	1-3 years	12710	0.86	0.83	0.90	< 0.001
	
	3-5 years	18303	0.88	0.85	0.91	< 0.001
	
	5-7 years	11251	0.88	0.84	0.93	< 0.001
	
	≥ 7 years	5280	0.97	0.90	1.05	0.450
	
	Year					
	
	2000	7066	0.83	0.78	0.88	< 0.001
	
	2001	7240	0.80	0.75	0.85	< 0.001
	
	2002	7369	0.88	0.82	0.93	< 0.001
	
	2003	7435	0.90	0.85	0.95	< 0.001
	
	2004	7494	0.91	0.86	0.96	0.001
	
	2005	7445	0.93	0.87	0.99	0.014
	
	2006	7357	0.91	0.85	0.97	0.002
	
	Children age					
	
	0-12 months	2980	0.92	0.81	1.04	0.191
	
	13-24 months	3620	0.94	0.86	1.03	0.166
	
	25-36 months	4027	0.88	0.81	0.96	0.003
	
	37-48 months	3970	0.91	0.83	1.00	0.059
	
	49-60 months	3385	0.89	0.80	1.00	0.040

The multivariate logistic regression model that considers tubewell access, flood control, population density and female adult education (or wealth index) simultaneously as independent variables also shows that greater tubewell access was associated with less childhood diarrhea (OR = 0.87, 95% CI: 0.84-0.89) (Table 2). In addition, flood control and education were negatively associated with childhood diarrhea, while population density was positively associated with childhood diarrhea.

**Table 2:** Associations between childhood diarrhea and tubewell access and depth inferred from multivariate logistic regressions

No. analyses	Independent variables	n	OR	95% CI		p
1.	Tubewell access	51406	0.87	0.84	0.89	< 0.001
			
	Flood control		0.82	0.78	0.85	< 0.001
			
	Population density		1.20	1.17	1.23	< 0.001
			
	Education		0.94	0.93	0.96	< 0.001

2.	Tubewell access	51406	0.87	0.85	0.89	< 0.001
			
	Flood control		0.84	0.80	0.87	< 0.001
			
	Population density		1.19	1.16	1.22	< 0.001
			
	Wealth		0.89	0.87	0.91	< 0.001

3. (Intermediate-depth wells)	Tubewell depth^a^	45600	1.25	1.20	1.31	< 0.001
			
	Flood control		0.77	0.74	0.80	< 0.001
			
	Population density		1.15	1.12	1.18	< 0.001
			
	Education		0.93	0.91	0.95	< 0.001

4. (Intermediate-depth wells)	Tubewell depth^a^	44975	1.26	1.21	1.31	< 0.001
			
	Flood control		0.78	0.75	0.82	< 0.001
			
	Population density		1.15	1.12	1.17	< 0.001
			
	Wealth		0.88	0.86	0.90	< 0.001

5. (Deep wells)	Tubewell depth^b ^	29900	0.91	0.77	1.06	0.220
			
	Flood control		0.70	0.67	0.74	< 0.001
			
	Population density		1.16	1.12	1.19	< 0.001
			
	Education		0.94	0.92	0.96	< 0.001

6. (Deep wells)	Tubewell depth^b ^	29425	0.90	0.77	1.06	0.2017
			
	Flood control		0.72	0.69	0.76	< 0.001
			
	Population density		1.15	1.12	1.19	< 0.001
			
	Wealth		0.89	0.87	0.92	< 0.001

a. Tubewell depth includes two categories: shallow wells and intermediate-depth well. b. Tubewell depth includes two categories: shallow wells and deep wells. For both cases, shallow wells are used as a reference.

The No. 1, 3 and 5 analyses are similar as the No. 2, 4 and 6 analyses, respectively, except that the education variable is replaced with the wealth variable.

### Associations between childhood diarrhea and tubewell depth

The analysis of diarrheal disease and depth shows that intermediate-depth wells (140-300 feet) were associated with more childhood diarrhea than shallow wells (less than 140 feet) over the entire study period (OR = 1.24, 95% CI: 1.19-1.29) (Table 3). The association also holds for individual years (ORs = 1.15-1.35). The associations between tubewell depth and childhood diarrhea were adjusted separately by the five control variables, flood control, wealth, education, population density and the child's age. Whether in a flood-controlled area or not, intermediate-depth wells were associated with a significantly higher number of cases of childhood diarrhea than shallow wells (OR > 1.00, p < 0.001). This was also true in each category of population density and education. The association between diarrheal disease and well depth was only altered in the poorest (wealth = 1) and the richest (wealth = 5) *baris*. For these two categories, intermediate-depth wells were not associated with a significantly higher number of cases than shallow wells (OR > 1, p > 0.05). The association of intermediate-depth wells with a higher prevalence of diarrhea than shallow wells held in *baris *with children aged 13-48 months but was altered in *baris *with children aged 0-12 months or 49-60 months.

**Table 3:** Comparison of the odds ratio of diarrhea risk between drinking intermediate-depth tubewell and shallow tubewell water

Comparison	Control variables	n	OR	95%CI		p
Intermediate-depth wells vs. shallow wells	Unstratified	45600	1.24	1.19	1.29	< 0.001
	
	Flood control					
	
	Yes	17926	1.43	1.33	1.53	< 0.001
	
	No	33480	1.15	1.09	1.21	< 0.001
	
	Population density					
	
	Low	13157	1.25	1.16	1.36	< 0.001
	
	Medium	15191	1.22	1.14	1.31	< 0.001
	
	High	17252	1.25	1.17	1.33	< 0.001
	
	Wealth					
	
	Low	2510	1.13	0.94	1.35	0.197
	
	Low medium	11991	1.28	1.18	1.38	< 0.001
	
	Medium	19605	1.29	1.22	1.37	< 0.001
	
	High medium	9172	1.22	1.12	1.34	< 0.001
	
	High	1697	1.26	0.99	1.59	0.056
	
	Education					
	
	< 1 year	3513	1.21	1.04	1.42	0.017
	
	1-3 years	11417	1.20	1.11	1.30	< 0.001
	
	3-5 years	16639	1.33	1.25	1.43	< 0.001
	
	5-7 years	9895	1.25	1.14	1.36	< 0.001
	
	≥ 7 years	4136	1.28	1.11	1.48	0.001
	
	Year					
	
	2000	6279	1.30	1.17	1.44	< 0.001
	
	2001	6436	1.30	1.17	1.45	< 0.001
	
	2002	6540	1.15	1.03	1.28	0.011
	
	2003	6597	1.17	1.05	1.30	< 0.004
	
	2004	6641	1.16	1.05	1.29	0.005
	
	2005	6590	1.35	1.21	1.50	0.001
	
	2006	6517	1.34	1.20	1.50	0.001
	
	Children age					
	
	0-12 months	2639	1.03	0.82	1.31	0.775
	
	13-24 months	3198	1.18	1.01	1.38	0.036
	
	25-36 months	3605	1.34	1.15	1.56	< 0.001
	
	37-48 months	3486	1.39	1.18	1.63	< 0.001
	
	49-60 months	3015	1.11	0.91	1.35	0.300

*Baris *drinking shallow tubewell water are the referent group. Univariate logistic regressions were used.

Deeper wells (≥ 300 feet) were associated with a lower prevalence of diarrhea compared with shallow wells in almost every year, although the relationships were not statistically significant (Table 4). Deep wells (≥ 300 feet) were not significantly associated with a change in prevalence of diarrhea compared to shallow wells (OR < 1.00, p > 0.05) when controlling for wealth. This result was not affected by whether *baris *were in the flood controlled area or outside. For population density and education in the lowest category, deep wells were associated with a lower risk of childhood diarrhea than shallow wells (OR < 1.00 and p < 0.05). Similar results were obtained using multivariate logistic regression models (Table 2).

**Table 4:** Comparison of the odds ratio of diarrhea risk between drinking deep tubewell and shallow tubewell water

Comparison	Control variables	n		OR	95% CI	p
Deep wells vs. shallow wells	Unstratified	29900	0.86	0.74	1.01	0.063
	
	Flood control					
	
	Yes	11088	0.81	0.63	1.05	0.118
	
	No	18812	0.92	0.76	1.13	0.430
	
	Population density					
	
	Low	8599	0.70	0.51	0.96	0.028
	
	Medium	10407	0.94	0.72	1.23	0.670
	
	High	10894	0.94	0.73	1.21	0.647
	
	Wealth					
	
	Low	1789	1.52	0.83	2.77	0.179
	
	Low medium	5468	0.75	0.51	1.10	0.142
	
	Medium	12846	0.95	0.76	1.20	0.668
	
	High medium	5395	0.81	0.58	1.12	0.206
	
	High	1109	0.71	0.29	1.72	0.446
	
	Education					
	
	< 1 year	2471	0.11	0.02	0.82	0.031
	
	1-3 years	7989	1.22	0.88	1.68	0.236
	
	3-5 years	10819	0.78	0.57	1.05	0.105
	
	5-7 years	6424	0.96	0.74	1.24	0.763
	
	≥ 7 years	3197	0.58	0.32	1.05	0.072
	
	Year					
	
	2000	4118	0.79	0.51	1.22	0.292
	
	2001	4227	0.85	0.55	1.31	0.462
	
	2002	4290	0.84	0.56	1.27	0.410
	
	2003	4335	0.83	0.56	1.26	0.385
	
	2004	4352	0.94	0.64	1.39	0.755
	
	2005	4316	0.79	0.51	1.22	0.286
	
	2006	4262	1.13	0.74	1.72	0.573
	
	Children age					
	
	0-12 months	1731	0.46	0.14	1.49	0.194
	
	13-24 months	2097	0.50	0.25	0.99	0.046
	
	25-36 months	2362	1.08	0.62	1.90	0.777
	
	37-48 months	2261	0.75	0.36	1.53	0.426
	
	49-60 months	1968	0.68	0.26	1.73	0.413

*Baris *drinking shallow tubewell water are the referent group. Univariate logistic regressions were used.

## Discussion

Millions of tubewells have been installed throughout Bangladesh over the past several decades, for the most part privately by individual households, in order to access what was widely believed to be safe drinking water. Although diarrhea morbidity has dropped considerably over the past several decades in Bangladesh, studies contemporary with the introduction of tubewells could not show that tubewell users were less affected by cholera or non-cholera diarrhea than non-tubewell users [7-9].

One difficulty in studying the relationship between drinking water quality and diarrhea lies in the uncertainty of exposure to different types of water. To overcome this problem, we created here an index of tubewell access based on tubewell density within neighborhoods around extended households. The underlying assumption is that people living in *baris *with a higher local-level tubewell density have better access than people in *baris *with a lower tubewell density. One reason this could have an impact on diarrhea is that people with greater access to tubewells can more easily maintain personal hygiene. This has been shown by lower bacteria counts measured on the hands of women in Bangladesh who were provided hand pumps and pit latrines compared to a control group [20]. Households with less access to a tubewell may also store water for longer periods of time, thus increasing the chance of microbial contamination of their drinking water [21].

Regardless of exactly why diarrhea morbidity has declined over the past several decades as a result of tubewell installation [6], our results based on the collection of childhood diarrhea cases for a large population clearly indicate that greater access to tubewells had a positive impact over the 7-year study period. The implication is that the introduction of tubewells several decades ago probably also helped reduce childhood diarrhea, along with other interventions to improve sanitation and hygiene [22,23].

These findings are consistent with a meta- analysis conducted by Fewtrell et al. suggesting that water, sanitation and hygiene interventions had similar effects on the reduction of diarrheal diseases in developing countries, with relative risk estimations ranging between 0.63 to 0.75 [24]. Another meta-analysis conducted by Clasen et al. suggested that interventions to improve water quality are generally effective for preventing diarrhea in all ages but the reason is unclear [25]. It is also unclear whether the decline in diarrheal disease recorded in Matlab between 2003 and 2006 was part of a long-term trend or driven by other factors that are beyond the scope of this study.

The observation that tubewell access is more important for reducing childhood diarrhea in areas of Matlab that are not flood controlled has policy implications, especially since access has the biggest impact for poorer people in flood-prone areas. Natural disasters such as flooding have been shown to increase the risk of diarrheal disease [26]. The lack of a significant relationship between tubewell access and diarrhea within the embankment suggests that the protective effect of flood control on diarrheal diseases overshadows the protective effect of a nearby tubewell. The significance of the relationship between access and diarrhea outside the embankment, on the other hand, suggests that installation of additional tubewells in areas without flood control where access is limited would likely further reduce diarrheal diseases.

Our finding that intermediate-depth tubewells are associated with an increase in diarrheal disease is surprising. In principle, the penetration of pathogens discharged by latrines and ponds into aquifers should decline with depth because of retention by the sediment. One possible explanation for the increase in diarrhea associated with intermediate-depth wells relates to the volume of water pumped from these household wells. Households using a private well typically pump 20-60 liters over the course of a day based on a household typically having 2-6 persons and a domestic water demand of 10 liters per person per day [27]. This corresponds to a proportion of the standing volume of water within a well that decreases from 1-3 well volumes daily for a 60-foot well to only a 0.2-0.6 well volume for a 300-foot well, assuming a standard 1.5" diameter PVC pipe was used to construct a well. If pathogens can reach the standing water within a well either from above or alongside the outside of a poorly sealed well and grow within the well [28], more effective flushing of a shallow well compared to an intermediate-depth well could potentially reduce the level of pathogens in drinking water and therefore reduce diarrheal disease. The association between intermediate-depth wells and childhood diarrhea could also potentially have resulted from the geographic distribution of the depth of tubewells or a confounding variable associated with local geology (Figure 1). However, spatial scan statistics show that the area where intermediate-depth wells are concentrated is not the area where childhood diarrhea cases are clustered [29].

The association between diarrhea and intermediate-depth wells is not confounded by flood control, wealth, or education even though both are associated with childhood diarrhea. The three variables are independent of one another and there is no significant difference in flood control or wealth between shallow and intermediate-depths. There is also no statistical difference in the proportion of wells in the three depth categories within and outside the embankment and the same is true for wealth and education. It is worth noting that the relationship between tubewell depth (intermediate-depth wells) and diarrhea has different odds ratios in the presence of flood control (OR = 1.43) in contrast to the absence of flood control (OR = 1.15). According to the 95% confidence intervals, these two odds ratios are heterogeneous, which is attributable to the interaction effect between flood control and tubewell depth on childhood diarrhea (details not shown). Even so, an unidentified confounder cannot be ruled out entirely.

Our finding concerning increased diarrhea associated with intermediate-depth wells may have implications for arsenic mitigation. Response surveys to well testing for As have shown that approximately one-third of the population of Bangladesh exposed to high levels of As has switched to a low-arsenic well [30]. Unlike some other areas of Bangladesh, the majority of these low-As wells are of intermediate depth in Matlab [31]. Diarrheal disease could therefore have increased in Matlab as a result of As mitigation by switching to intermediate-depth wells

Our analysis does not indicate a robust relationship between the prevalence of diarrheal disease for households and usage of the deepest category of wells, which are typically also low in As in Matlab. A more detailed study conducted in a limited number of Matlab villages has shown, moreover, that childhood diarrhea declined more rapidly in households that switched to deep wells compared to households that continued to use a shallower well [32]. These observations do not necessarily contradict the relationship between well depth and flushing invoked to explain increased diarrheal disease associated with intermediate-depth wells. Deep wells are typically community wells installed by the government and NGOs, and are used by a much larger number of household pumping 500-1000 liters each day [33]. The rate of flushing of deep community wells is therefore comparable to that of shallow private wells.

## Conclusions

This study shows that greater access to tubewells is associated with significantly lower prevalence of diarrheal disease in children. This finding suggests that drinking tubewell water still protects children from diarrheal diseases and that the installation of tubewells should not be discouraged. However, our results indicate that drinking intermediate-depth wells is associated with a higher risk of diarrhea than drinking shallow or deep wells, which is surprising. The origin of the greater risk of diarrheal disease associated with intermediate-depth tubewells needs to be investigated further.

## List of abbreviations

As: Arsenic; CI: Confidence interval; CHRW: Community health research workers; GIS: Geographic Information System; GPS: Global Positioning System; HDSS: Health and Demographic Surveillance System; ICDDR; B: International Centre for Diarrhoeal Disease Research; Bangladesh; OR: Odds ratio; WHO: World Health Organization.

## Competing interests

The authors declare that they have no competing interests.

## Authors' contributions

JW, AvG and ME participated in the design of the study, performed the statistical analysis, and drafted the manuscript. MY and PKS participated in the acquisition of health data. MY, VE YA and MS participated in study conceiving and design. All authors read and approved the final manuscript.
